# Cognitive behavioral therapy program for cannabis use cessation in first-episode psychosis patients: study protocol for a randomized controlled trial

**DOI:** 10.1186/s13063-016-1507-x

**Published:** 2016-07-29

**Authors:** Itxaso González-Ortega, Enrique Echeburúa, Adriana García-Alocén, Patricia Vega, Ana González-Pinto

**Affiliations:** 1 Center for Biomedical Research in the Mental Health Network (CIBERSAM), Madrid, Spain; 2 Department of Psychiatry, Araba University Hospital, Olaguibel Street 29, 01004 Vitoria, Spain; 3 School of Psychology, University of the Basque Country, San Sebastián, Spain; 4 School of Medicine, University of the Basque Country, Vitoria, Spain

**Keywords:** Cannabis, First-episode psychosis, Psychological treatment

## Abstract

**Background:**

The high rate of cannabis use among patients with first-episode psychosis (FEP), as well as the associated negative impact on illness course and treatment outcomes, underlines the need for effective interventions in these populations. However, to date, there have been few clinical treatment trials (of pharmacological or psychological interventions) that have specifically focused on addressing comorbid cannabis use among these patients. The aim of this paper is to describe the design of a study protocol for a randomized controlled trial in which the objective is to assess the efficacy of a specific cognitive behavioral therapy program for cannabis cessation in patients with FEP compared to standard treatment (psychoeducation).

**Methods/design:**

This is a single-blind randomized study with 1 year of follow-up. Patients are to be randomly assigned to one of two treatments: (1) specific cognitive behavioral therapy for cannabis cessation composed of 1-hour sessions once a week for 16 weeks, in addition to pharmacological treatment scheduled by the psychiatrist, or (2) a control group (psychoeducation + pharmacological treatment) following the same format as the experimental group. Participants in both groups will be evaluated at baseline (pre-treatment), at 16 weeks (post-treatment), and at 3 and 6 months and 1 year of follow-up. The primary outcome will be that patients in the experimental group will have greater cannabis cessation than patients in the control group at post-treatment. The secondary outcome will be that the experimental group will have better clinical and functional outcomes than the control group.

**Discussion:**

This study provides the description of a clinical trial design based on specific cognitive behavioral therapy for cannabis cessation in FEP patients, aiming to improve clinical and functional outcome, as well as tackling the addictive disorder.

**Trial registration:**

NCT02319746 ClinicalTrials.gov Identifier. ClinicalTrials.gov Protocol and Results Registration System (PRS) Receipt Release Date: 15 December 2014.

**Electronic supplementary material:**

The online version of this article (doi:10.1186/s13063-016-1507-x) contains supplementary material, which is available to authorized users.

## Background

Substance misuse is a common comorbid problem in patients with first-episode psychosis (FEP), cannabis being the most commonly abused substance together with alcohol in this population [1]. The prevalence of cannabis use among patients with FEP is notably high, at around 65.7% [2]. This has led to the hypothesis that its use may have a role in the etiology and evolution of psychosis. It has been found that cannabis use may act as a risk factor influencing age of onset of psychosis [3–8]. Further, young people who abuse cannabis have a higher risk of developing psychosis than non-abusers, with an earlier age of onset of psychosis [9]. The risk of developing psychosis is also related to an early age of onset of use [3, 9–11], and to the severity of use [3, 9], with a frequency- and dose-dependent response, especially in vulnerable individuals [3, 12], increasing the risk in the general population.

Recently, it has also been suggested that cannabis abuse in adolescence can cause alterations in the endocannabinoid system (ECS) and that these alterations may be related to a higher incidence of psychosis and to some of the symptoms presented. Specifically, some research has shown that frequent use of cannabis can cause inhibition of the signaling pathways of the main endocannabinoid, anandamide, in schizophrenia patients but not in healthy individuals [13]. It has also been reported that FEP patients who are cannabis users have cognitive deficits associated with structural abnormalities of brain areas with a high level of expression of cannabinoid receptor type 1 (CB1) [14, 15]. Further, it has been shown that the intake of exogenous cannabinoids could alter synaptic plasticity mediated by the ECS, possibly affecting brain maturation during adolescence, and in turn, neurodevelopmental processes [16].

The ECS is an endogenous homeostatic system with multiple physiological functions and is activated in response to various different stimuli and under different neuropathological conditions. It is composed of a series of lipid mediators, known as endocannabinoids, anandamide and 2-arachidonoylglycerol being the most studied; their two classical receptors coupled to G protein, CB1 and cannabinoid receptor type 2 (CB2); and enzymes responsible for its synthesis, *N*-acyl-phosphatidylethanolamine-selective phospholipase C (NAPE-PLC) and diacylglycerol lipase (DAG-L), and degradation, fatty acid amide hydrolase (FAAH) and monoacylglycerol lipase (MAG-L).

Some studies have linked the ECS with psychotic illness, especially focusing on CB1 and CB2. In particular, lower expression of CB1 has been observed in several brain regions of individuals with schizophrenia [17] and it has also been shown that CB2 loss of function (due to polymorphisms such as *Q63R*) is associated with an increased susceptibility to schizophrenia [18]. Remission of symptoms in schizophrenia has also been associated with significant changes in messenger ribonucleic acid (mRNA) levels of CB2 in peripheral blood mononuclear cells [19]. With respect to other components of the ECS, there is much less information, but alterations in the levels of anandamide have been found in the cerebrospinal fluid of patients with schizophrenia [20].

There is empirical evidence for an influence of cannabis on the course and outcome of psychosis [21, 22]. Cannabis use in early psychosis is correlated with poor adherence to pharmacological treatment [2, 23, 24], the severity of psychotic symptoms [22] and the risk of relapse [2, 21, 23, 24]. Moreover, patients with FEP who use cannabis have a poorer functional outcome at follow-up [22, 25–27]. On the other hand, patients with FEP who stop using cannabis experience a slow but steady improvement over time in clinical and functional outcome [21].

Cannabis use, due to its influence on the development and prognosis of the disease, has become a target for the prevention and treatment of patients with FEP. Such patients, unlike chronic psychotic patients, show a greater variation in the frequency and intensity of cannabis use and greater motivation for change at baseline [28]. Therefore, it is essential to intervene in the early stages of the disease, before cannabis use becomes established. What is more, early intervention may be able not only to reduce but also to detect this type of substance abuse in patients with FEP.

In a systematic review, Ruiz de Azúa García et al. [29] concluded that several studies have shown that psychoeducation as an adjuvant to pharmacological treatment is effective in improving negative symptoms and functionality (Lambeth Early Onset Team Study, UK; Personal Assessment and Crisis Evaluation, Australia; OPUS Scandinavia, Denmark; Early Psychosis Prevention and Intervention Centre, Australia). Evidence suggests that cognitive behavioral therapy may also be effective in FEP [30]. However, despite the recognized clinical consequences of cannabis use in early psychosis [3–6], only a few trials have evaluated clinical treatments as a specific intervention to reduce cannabis use in this population. These studies have investigated interventions based on motivational interviewing [31] or combined interventions based on motivational interviewing and cognitive behavioral therapy [32–35]. Generally, the results obtained failed to clearly indicate whether the interventions were effective in terms of reducing cannabis use and/or improving clinical outcomes at follow-up. The exception was the study conducted by Madigan et al. [34], in which patient quality of life improved at post-treatment. Further, the small sample sizes and the absence of an appropriate treatment-as-usual control condition in these studies indicate that it is not possible to draw definite conclusions.

From reviewing the literature, we conclude that there is an urgent need to develop effective specific interventions to reduce cannabis use as part of comprehensive treatment programs for patients with psychosis. Overall, the effectiveness of interventions and the type of approach used are unclear [31–35]. There is also a need for interventions based on more precise knowledge of individual differences in patterns of use as well as of factors that maintain or inhibit substance abuse in young people with FEP.

The main aim of this article is to describe the design of a randomized controlled trial focused on comparing the efficacy of a specific cognitive behavioral therapy program for cannabis cessation with standard treatment in patients with FEP who are cannabis users. The study design, assessment and intervention program are described.

The specific objectives of the study are:

1. To assess whether a specific cognitive behavioral therapy program for cannabis cessation is associated with a greater reduction in use of cannabis than standard treatment
2. To assess whether this type of program for cannabis cessation is associated with better outcomes of the psychotic disorder (i.e., reduction in symptoms and improvement in psychosocial functioning) in the follow-up than standard treatment
3. To analyze the relation between cannabis abstinence and clinical and functional outcomes of patients
4. To determine whether there are systemic alterations in the components of the ECS and, if so, whether a specific cognitive behavioral therapy program for cannabis cessation is able to normalize such alterations
5. To assess whether normalization of elements of the ECS is directly related to reductions in symptoms and improvements in psychosocial functioning attributable to the proposed specific treatment

## Methods

### Design

This is a randomized study with 1 year of follow-up. The intervention programs will be offered at Araba University Hospital and biological samples will be analyzed at Complutense University of Madrid.

The randomized clinical trial was registered in 2014 (ClinicalTrials.gov Identifier NCT02319746). This clinical trial fulfills the Standard Protocol Items: Recommendations for Interventional Trials (SPIRIT) Checklist (Additional file 1). The efficacy of a specific treatment program for cannabis abuse (cognitive behavioral treatment + pharmacological treatment) will be compared to that of standard treatment (psychoeducation + pharmacological treatment) in patients with FEP who are cannabis users.

### Participants

FEP patients who are cannabis users and meet the inclusion criteria (listed below) are to be included and randomly assigned to one of the two treatment groups. The sample size calculation was performed using data published in the literature related to the main theme of the study (Bonsack et al., 2011 [31]; Edwards et al., 2006 [32]; Hjorthøj et al., 2012; Madigan et al., 2013 [34]) and using Ene 2.0 software. To achieve a power of 80% to detect differences from the null hypothesis, H0: *μ*1 = *μ*2, using a bilateral Student’s *t* test for two independent samples, with a significance level of 5%, we need to include 30 patients in the experimental group and 30 patients in the control group, meaning a total of 60 patients for the study.

#### Inclusion criteria

The study inclusion criteria for both groups are:

1. Being diagnosed as having had a first psychotic episode: (i.e., schizophrenia, schizophreniform disorder, schizoaffective disorder, delusional disorder, bipolar disorder, atypical psychosis, brief psychotic disorder, or major depressive disorder with psychotic symptoms) according to the revised fourth edition of *Diagnostic and Statistical Manual of Mental Disorders, Fourth Edition, Text Revision* (DSM-IV-TR) [36]
2. Being a regular cannabis user (Table 1):

**Table 1:** Classification of cannabis use for selection of participants

Severity of consumption	DSM-IV-TR^a^ criteria for abuse or dependence	Europ-ASI^b^ scores
Dependence	Meet at least minimal DSM-IV-TR criteria for cannabis dependence	8–9
Abuse	Meet ≥1 DSM-IV-TR criteria for cannabis abuse	4–7
Use	Meet DSM-IV-TR criteria for cannabis abuse but not the duration criterion (≥12 months) or ≥12 months of use but do not meet any DSM-IV-TR criteria for cannabis abuse	2–3
No use	No significant symptoms	0–1

^a^Revised Fourth Edition of the Diagnostic and Statistical Manual of Mental Disorders (DSM-IV-TR) [37]

^b^European Addiction Severity Index (Europ-ASI) [37,38]
  Dependence or abuse of cannabis according to *Diagnostic and Statistical Manual of Mental Disorders, Fourth Edition, Text Revision* (DSM-IV-TR) criteria [36].
  Dependence or abuse of cannabis according to the scores of the European Addiction Severity Index (EUROP-ASI) [37, 38] (scores of 4 to 7: abuse; scores of 8 to 9: dependence)
3. Aged between 15 and 40 years. In the case of minors (under 18 years of age), written informed consent will be requested from their parents or guardians
4. Being in remission from a first psychotic episode (the patients being required to be in remission, without any relapses in a period no more than 5 years from the first psychotic episode)

#### Exclusion criteria

The study exclusion criteria for both groups are:

1. Presenting organic brain pathology
2. Presenting mental retardation according to DSM-IV-TR criteria

#### Randomization

Patients are to be randomly assigned to one of the two treatment groups by permuted block randomization with a block size of 4 and a 1:1 allocation using a computer-generated random sequence. The allocation sequence will be prepared by an independent person not otherwise involved in the trial.

### Assessment

Data collection is to be based on an assessment protocol for gathering data on sociodemographic, clinical and cannabis/other substance use-related variables. All patients are to be assessed at baseline, post-treatment and in the follow-up period (at 3 and 6 months and 1 year of follow-up from the end of the treatment program) (Table 2).

**Table 2:** Assessment protocol

Assessments	Pre-treatment	Post-treatment	Follow-up^a^		
Sociodemographic variables	X				
Cannabis/other substance use-related variables					
Use	X	X	X	X	X
Severity	X	X	X	X	X
Clinical variables					
Diagnosis	X	X	X	X	X
Clinical severity	X	X	X	X	X
Illness awareness	X	X	X	X	X
Medication adherence	X	X	X	X	X
Clinical symptomatology	X	X	X	X	X
Psychosocial functioning	X	X	X	X	X
Biological variables					
Peripheral blood mononuclear cells	X	X	X	X	X
Toxins in urine	X	X	X	X	X

^a^Follow-up: 3 and 6 months and 1 year of follow-up from the end of the intervention program

#### Sociodemographic variables

Data on sociodemographic variables (age, gender, educational level, socioeconomic level, employment status, family history of psychiatric disorders) are to be collected at baseline.

#### Cannabis/other substance use-related variables

1. *Variables related to cannabis/other substance use* include frequency of use, dose, age of onset of use and history of use (years)
2. *Severity of cannabis/other substance use* is assessed with the EUROP-ASI [37, 38] and the Cannabis Use Problems Identification Test (CUPIT) [39]

The EUROP-ASI, an adaptation of the Addiction Severity Index (fifth version), is a structured interview for clinical practice and research. It is designed to assess severity of the substance use problem and makes it possible to monitor and quantify changes in problems commonly associated with substance abuse. Translated into practically all European languages, the reliability and validity of EUROP-ASI are well established [40–44] and it has been shown to have high internal consistency (Cronbach’s alpha coefficient >0.70) [41, 42, 44, 45] and moderate-excellent interrater reliability (interclass correlation coefficient 0.62–0.99) [43, 44].

CUPIT is a brief self-report screening instrument for the detection of currently and potentially problematic cannabis use. It is composed of 16 items and has demonstrated good test-retest (0.88 to 0.99) and internal consistency reliabilities for the two derived subscales, “dependence” (0.92, whole sample) and “problems” (0.90 adults, 0.79 adolescents), and it has reliably discriminated diagnostic subgroups (no diagnosis, abuse/harmful use, dependence) across the problem severity continuum (diagnostic utility) [39].

#### Clinical variables

1. Diagnosis
  Patients are diagnosed according to the DSM-IV-TR criteria using the Structured Clinical Interview for the Diagnostic and Statistical Manual of Mental Disorders, Axis I Disorders (SCID-I) [45]. This interview is to be carried out independently but at the same time by two experienced clinicians, to confirm inter-rater reliability in the diagnosis of patients
2. Clinical severity
  The Clinical Global Impression Scale (CGI) [46] is used to assess symptom severity, global improvement and therapeutic response. It is a 3-item observer-rated scale. Items 1 and 2 are rated on a seven-point scale; and item 3 is rated on a five-point scale, from 0 to 4 (taking into account therapeutic efficacy and treatment-related adverse events)
3. Illness awareness
  The illness awareness of patients is measured using the Scale to assess Unawareness in Mental Disorders (SUMD) [47, 48]. This scale explores the thoughts and beliefs of patients regarding their illness and its pharmacological treatment
4. Medication adherence
  The type of pharmacological treatment is recorded and medication adherence is estimated with the 4-item Morisky Medication Adherence Scale [49, 50]. It assesses attitudes of patients towards their treatment. Patients with a score of 4 were considered to have good adherence, while those with a score between 0 and 3 were classified as having poor adherence
5. Clinical symptomatology
  Psychotic symptoms are measured using Positive and Negative Syndrome Scale (PANSS) [51, 52]. The PANSS is a relatively brief interview used for measuring positive, negative and general symptoms of patients with schizophrenia. The ratings provide summary scores on a 7-item positive scale, a 7-item negative scale and a 16-item general psychopathology scale.
  Depressive symptoms are measured with the Hamilton Depression Rating Scale (HDRS-21) [53, 54]. This is a 21-item scale that assesses the severity of depressive symptoms (range: 0–52)
  Manic symptoms are measured using the Young Mania Rating Scale (YMRS) [55, 56]. This is an 11-item scale used to assess the severity of manic symptoms (range: 0–60).
  Anxiety symptoms are measured using the Hamilton Anxiety Scale (HAM-A) [57, 58]. This has been developed to measure the severity of anxiety symptoms, both psychic anxiety (mental agitation and psychological distress) and somatic anxiety (physical complaints related to anxiety)
6. Psychosocial functioning
  The functioning of patients is measured using the Functioning Assessment Short Test (FAST) [59]. The FAST is a brief instrument designed to assess functional impairment in severe mental disorders. The 24 items of the scale cover six specific areas of functioning: autonomy, occupational functioning, cognitive functioning, financial issues, interpersonal relationships and leisure time

#### Biological variables

To assess whether the treatment program is able to normalize any changes in the components of the ECS, peripheral blood mononuclear cells (10 ml of venous blood anticoagulated with ethylenediaminetetraacetic acid (EDTA)) are to be collected at baseline, at post-treatment and at 3 and 6 months and 1 year of follow-up. In peripheral blood mononuclear cells, the following elements of the endogenous cannabinoid system are to be measured: synthesis enzymes (NAPE-PLD, DAG-L), degrading enzymes (FAAH, MAG-L), and receptors (CB1 and CB2) by Western blot (protein) and reverse transcription-polymerase chain reaction (mRNA).

Toxins in urine are assessed with an immunochromatographic test to detect drug metabolites at baseline, at sessions 4 and 8 of the psychological treatment, post-treatment, and at 3 and 6 months and 1 year of follow-up.

#### Procedure

**Fig. 1:**
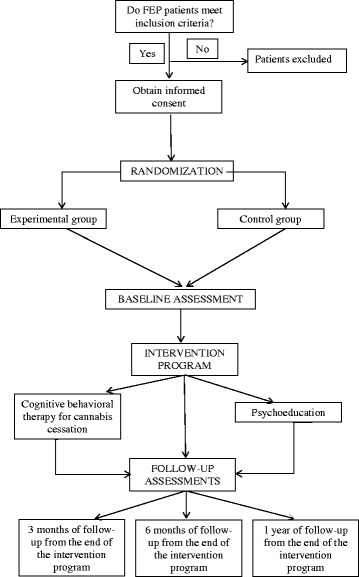
Study procedure

Patients will be assessed after being informed of the objectives of the study and giving their informed consent to participate. All participants will be evaluated individually and will be randomly assigned to one of two treatment groups. Data will be collected following an assessment protocol (see “Assessment” section) that will be implemented at baseline, post-treatment and in the follow-up period (at 3 and 6 months and 1 year of follow-up from the end of the intervention program) (Fig. 1).

#### Statistical analysis

IBM SPSS version 23 will be used for the statistical analysis. The sociodemographic characteristics of the patients will be analyzed using descriptive statistics. Differences between the two intervention groups (cognitive behavioral therapy versus psychoeducation) will be analyzed using the χ^2^ test (or Fisher’s test where *n* ≤5) for categorical variables and the Student’s *t* test or Mann-Whitney *U* test (depending on the distribution of the data) for continuous variables.

The differences in cannabis use reduction between two intervention groups will be analyzed using Student’s *t* test and/or the Mann-Whitney *U* test and mixed models. Effect sizes will be calculated for differences in cannabis abstinence based on Cohen’s *d* or *r* [60] for Student’s *t* test and the Mann-Whitney *U* test, respectively. The results will be interpreted as small if *d* values are between .2 and .49, medium if *d* values are between .5 and .79, and large if *d* values are higher than .8.

Differences in efficacy between the interventions in terms of clinical and functional outcomes of patients will be analyzed with repeated measures models (baseline, post-treatment, 3 and 6 months and 1 year of follow-up) and logistic regressions.

Furthermore, whether cannabis abstinence is associated with better clinical and functional outcomes will be analyzed using linear regression models for continuous dependent variables, and Poisson regression models for categorical dependent variables.

Finally, the biological data of patients will be studied in conjunction with clinical data using multivariate analyses. Specifically, the possible alterations in the components of the ECS and the influence of the specific cognitive behavioral therapy program for cannabis cessation on the normalization of ECS will be analyzed using repeated measures models, mixed models or analysis of covariance. Further, the relation between normalization of elements of the ECS and improvement in symptoms and psychosocial functioning, attributable to the specific treatment, will be assessed by regression models.

### Intervention programs

Patients are to be randomized into two treatment groups:

1. The experimental group will receive specific cognitive behavioral therapy for cannabis cessation composed of 1-hour sessions once a week for 16 weeks, in addition to pharmacological treatment prescribed by the psychiatrist
2. The control group will receive the standard treatment (psychoeducation + pharmacological treatment) following the same format as the experimental group, that is, 1-hour sessions of psychoeducation once a week for 16 weeks, in addition to pharmacological treatment prescribed by the psychiatrist

1. Experimental group (specific cognitive behavioral therapy for cannabis cessation + pharmacological treatment)
  The intervention program is focused on cannabis cessation, identification of prodromes, improving illness awareness, adherence to treatment, psychosocial functioning and relapse prevention. The content of the sessions is as follows:
  Sessions 1–3: the first three sessions involve motivational interviewing [61], followed by brief psychoeducation focused on general information about cannabis and psychosis: (a) psychosis and substance use, (b) medication and treatment adherence, (c) awareness of the vulnerability, (d) recognition of symptoms, (e) healthy lifestyle, and (f) risk and protective factors.
  Sessions 4–8: the second part of the program is focused on commitment to change [62] and includes the following aspects:
    Behavioral therapy:
    o Anxiety management techniques
    o Stimulus control
    o In-vivo exposure therapy with response prevention, identifying triggers and beliefs that could lead to substance use and exacerbation of psychotic symptoms and exposure to such triggers
    Cognitive therapy:
    o Specific techniques for managing thoughts about the consumption and use of cannabis and other substances (craving/abstinence) and symptom management
    o Cognitive restructuring; identifying and refuting cognitive distortions
    o Training in problem solving
    o Training in social skills; assertiveness; skills to refuse drugs and changes in lifestyle.
  Sessions 10–12: the third part of the program includes a specific intervention for relapse prevention, focused on the identification of high-risk situations that could lead to maintenance of substance use and increased severity and chronicity of psychotic symptoms, as well as the teaching of coping skills for such situations
2. Control group (standard treatment: psychoeducation + pharmacological treatment)

The aim of the psychoeducation is to enable patients to understand and be able to manage their disease, providing them with tools and skills for symptom management, to avoid relapse and contribute to their well-being. Psychoeducation sessions include the following modules:

- Session 1: What is a first psychotic episode?
- Session 2: Awareness of vulnerability
- Session 3: Recognition of symptoms
- Sessions 4–5: Prevention of relapses: risk and protective factors
- Session 6: Adherence to treatment
- Session 7: Healthy lifestyle: sleep and sexuality
- Session 8: Healthy lifestyle: misuse of drugs
- Session 9: Anxiety management techniques I
- Session 10: Anxiety management techniques II
- Session 11: Social skills: assertive communication techniques
- Session 12: Problem-solving techniques

## Discussion

The randomized clinical trial described in this paper represents an innovation related to the development of therapeutic procedures based on evidence. All patients diagnosed with severe mental illness should be included early in a program of effective treatment. Specifically, the effective treatment of psychotic disorders in patients with comorbid substance abuse is an important goal in clinical practice. Mental health drug addiction services are often run in parallel to other health care services with different, sometimes conflicting, approaches and this further hinders the treatment of these patients, who may have difficulty integrating into standard treatments and require specific programs. Ideally, interventions for these patients should be tailored to their specific needs. However, this is often not possible given the dichotomy between mental health and substance abuse treatment providers. On the other hand, no clinical treatment trials conducted to date have shown any specific interventions to have effective results in terms of reducing cannabis use and/or improving clinical outcomes in the follow-up of this population.

The results of our study may have a significant impact on both prognosis and treatment, and may be useful for identifying patients who need early and continuous therapeutic interventions from the onset of their illness, in addition to specific interventions to tackle the impact of drug use. The results of this study should also help us to meet the social and health needs of this population, guiding the use of therapeutic resources that may be required early in treatment to decrease the severity of the psychopathology and improve the prognosis of patients. This kind of intervention is a promising therapeutic approach not only to treat psychosis, but also to discover and reduce substance abuse in individuals experiencing a first psychotic episode.

Finally, the design of early intervention strategies and the development of a clinical guideline setting out a specific treatment program for patients with FEP who are cannabis users would enable more efficient management of health resources. That is, besides enriching treatment programs currently available, this study will help to standardize this new treatment, thereby serving the professional community, and in turn, patients will benefit from the results of this research.

The limitations of this study are mostly related to typical limitations of this type of clinical trial with a longitudinal design, in particular, the sample size. Another limitation is that this type of patient with a dual diagnosis often has a lack of illness awareness, and poor insight and treatment adherence, and hence there may be difficulties in completion of the program and attendance to follow-up visits by participants. These factors should be taken into consideration in interpreting the efficacy of the therapy and the impact of intervention on the clinical and functional outcomes of patients.

### Trial status

Patient recruitment has not been completed at the time of submission.

## Abbreviations

CB1, cannabinoid receptor type 1; CB2, cannabinoid receptor type 2; CGI, Clinical Global Impression Scale; CUPIT, Cannabis Use Problems Identification Test; DAG-L, diacylglycerol lipase; DSM-IV-TR, *Diagnostic and Statistical Manual of Mental Disorders, Fourth Edition, Text Revision*; ECS, endocannabinoid system; EUROP-ASI, European Addiction Severity Index; FAAH, fatty acid amide hydrolase; FAST, Functioning Assessment Short Test; FEP, first episode psychosis; HAM-A, Hamilton Anxiety Scale; HDRS-21, Hamilton Depression Rating Scale; MAG-L, monoacylglycerol lipase; NAPE-PLC, *N*-acyl-phosphatidylethanolamine-selective phospholipase C; PANSS, Positive and Negative Syndrome Scale; SCID-I, Clinical Interview for the *Diagnostic and Statistical Manual of Mental Disorders*, Axis I Disorders; SUMD, Unawareness in Mental Disorders; YMRS, Young Mania Rating Scale

## Additional file

Additional file 1: SPIRIT Checklist. (DOC 122 kb)
